# Nonbilayer Phospholipid Arrangements Are Toll-Like Receptor-2/6 and TLR-4 Agonists and Trigger Inflammation in a Mouse Model Resembling Human Lupus

**DOI:** 10.1155/2015/369462

**Published:** 2015-10-19

**Authors:** Carlos Wong-Baeza, Alonso Tescucano, Horacio Astudillo, Albany Reséndiz, Carla Landa, Luis España, Jeanet Serafín-López, Iris Estrada-García, Sergio Estrada-Parra, Leopoldo Flores-Romo, Carlos Wong, Isabel Baeza

**Affiliations:** ^1^Biochemistry Department, National School of Biological Sciences, National Polytechnic Institute (IPN), 11340 Mexico City, DF, Mexico; ^2^Cell Biology Department, Center for Research and Advanced Studies of the National Polytechnic Institute (IPN), 07360 Mexico City, DF, Mexico; ^3^Oncology Department, Mexican Institute of Social Security, XXI Century National Medical Center, 06720 Mexico City, DF, Mexico; ^4^Immunology Department, National School of Biological Sciences, National Polytechnic Institute (IPN), 11340 Mexico City, DF, Mexico

## Abstract

Systemic lupus erythematosus is characterized by dysregulated activation of T and B cells and autoantibodies to nuclear antigens and, in some cases, lipid antigens. Liposomes with nonbilayer phospholipid arrangements induce a disease resembling human lupus in mice, including IgM and IgG antibodies against nonbilayer phospholipid arrangements. As the effect of these liposomes on the innate immune response is unknown and innate immune system activation is necessary for efficient antibody formation, we evaluated the effect of these liposomes on Toll-like receptor (TLR) signaling, cytokine production, proinflammatory gene expression, and T, NKT, dendritic, and B cells. Liposomes induce TLR-4- and, to a lesser extent, TLR-2/TLR-6-dependent signaling in TLR-expressing human embryonic kidney (HEK) cells and bone marrow-derived macrophages. Mice with the lupus-like disease had increased serum concentrations of proinflammatory cytokines, C3a and C5a; they also had more TLR-4-expressing splenocytes, a higher expression of genes associated with TRIF-dependent TLR-4-signaling and complement activation, and a lower expression of apoptosis-related genes, compared to healthy mice. The percentage of NKT and the percentage and activation of dendritic and B2 cells were also increased. Thus, TLR-4 and TLR-2/TLR-6 activation by nonbilayer phospholipid arrangements triggers an inflammatory response that could contribute to autoantibody production and the generation of a lupus-like disease in mice.

## 1. Introduction

Systemic lupus erythematosus (SLE) is a systemic autoimmune disease characterized by a loss of tolerance to nuclear antigens and by dysregulated activation of T and B cells. Polyclonal activation of B cells leads to the production of large quantities of autoreactive antibodies and the formation of immune complexes, which causes tissue damage. In some SLE patients, it has been shown that bone marrow mesenchymal stem cells exhibit impaired capacities for proliferation, differentiation, migration [1], and immune modulation [2]. Genetic defects, drug exposure, infectious agents, and environmental factors can also contribute to the pathogenesis of this disease [3, 4]. SLE has an incidence in Europe and North America of approximately 10 cases per 100,000 population per year, and it is estimated that 10% of these cases are drug-induced. Drug-induced lupus erythematosus (DILE) is a lupus-like syndrome that resolves upon drug discontinuation. The drugs more frequently associated with the induction of this lupus-like syndrome are procainamide (antiarrhythmic), hydralazine (antihypertensive), and chlorpromazine (antipsychotic) [5, 6].

**Figure 1:**
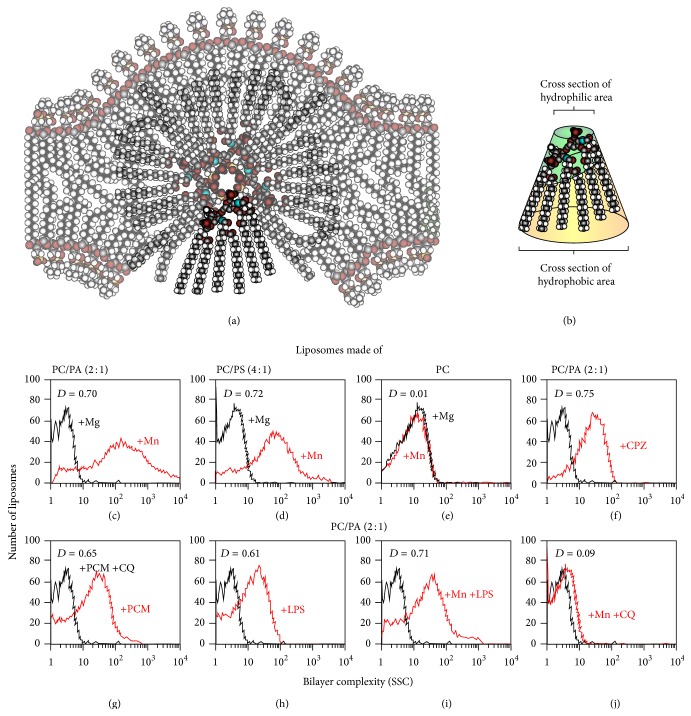
Structure and characterization of nonbilayer phospholipid arrangements. (a) Representation of a nonbilayer phospholipid arrangement, showing an inverted micelle, with the acyl chains of the phospholipids in a conical arrangement, inserted into the lipid bilayer. (b) Molecular shape of the lipid A of LPS, showing the conical arrangement of its acyl chains. (c to j) Liposomes made of egg-yolk phosphatidylcholine (PC)/egg-yolk phosphatidic acid (PA) (2 : 1 molar ratio), PC/bovine brain phosphatidylserine (PS) (4 : 1) (50 nmol anionic phospholipid in 50 *μ*L TS buffer), or PC alone were incubated at 37°C for 30 min with 2 mM MnCl_2_, 1.5 mM chlorpromazine (CPZ), 8 mM procainamide (PCM), 100 ng/mL LPS, 0.1 mM chloroquine (CQ), 0.2 mM MgCl_2_, or the indicated mixtures of cations or compounds, respectively. Changes in bilayer complexity (SSc) are represented as histograms: black lines represent liposomes alone or incubated with Mg or PCM + CQ; red lines represent liposomes incubated with Mn or the indicated compound. Values of Kolmogorov-Smirnov test of *D* ≥ 0.5, *p* < 0.001, indicate a significant difference between the compared histograms. A representative experiment of five is shown.

Animal models of SLE include lupus-prone mice, which spontaneously develop lupus, and normal mice that develop lupus after injection of lymphocytes from lupus-prone mice, immunization with prototypical lupus antigens (DNA- and RNA-protein complexes), or injection of pristane (2,6,10,14-tetramethylpentadecane) [3, 7]. The most commonly used lupus-prone mice are the F_1_ hybrids of New Zealand black (NZB) and NZ white (NZB/NZW F_1_) mice, the Murphy-Roths large/lymphoproliferative locus (MLR/lpr) mice, and the recombinant C57BL/6 female and SB/Le male strain/Y-linked autoimmune accelerator (BXSB/Yaa) mice [3, 8, 9]. Our group has also developed a mouse model of autoimmune disease resembling human lupus that can be induced in normal mice [10]. In this model, the disease is triggered by liposomes with nonbilayer phospholipid arrangements. Liposomes are model membranes made of cylindrical phospholipids, such as phosphatidylcholine, and H_II_-preferring (conical shaped) phospholipids, such as phosphatidic acid, phosphatidylserine, or cardiolipin [11]. Conical phospholipids can form molecular associations distinct to lipid bilayers, known as nonbilayer phospholipid arrangements, in the presence of inducers such as Mn^2+^ [12, 13] or the drugs chlorpromazine and procainamide, which can trigger DILE in humans [10]. Nonbilayer phospholipid arrangements are formed by an inverted micelle (made of conical phospholipids with their polar heads towards the center of the micelle, where the inducer is also located) inserted into and distorting the shape of the phospholipid bilayer (Figure 1(a)). We demonstrated that liposomes with nonbilayer phospholipid arrangements induced by Mn^2+^, chlorpromazine, or procainamide cause an autoimmune disease resembling human lupus in mice. A similar disease is produced by treating mice directly with Mn^2+^, chlorpromazine, or procainamide (which induce nonbilayer phospholipid arrangements on mouse cells) or by injecting the monoclonal antibody H308 (which binds specifically to nonbilayer phospholipid arrangements and stabilizes these arrangements on mouse cells) [10, 14].

IgM and IgG antibodies against nonbilayer phospholipid arrangements are found in the sera of mice with the autoimmune disease resembling human lupus, and also in the sera of patients with lupus [10, 15]. Usually, the efficient production of IgG antibodies requires an activation of the innate immune response. Therefore we hypothesized that nonbilayer phospholipid arrangements could be Toll-like receptor- (TLR-) 4/MD-2 agonists, as their molecular structure is similar to that of the lipid A from bacterial lipopolysaccharide (LPS). Lipid A is formed by a *β*-1,6-D-glucosamine disaccharide with two (negatively charged) phosphates and six saturated acyl chains in an asymmetric distribution (four chains are bound to the nonreducing and two to the reducing glucosamine). Hexaacylated asymmetric lipid A molecules have a conical molecular shape, because the cross section of the hydrophobic region is larger than that of the hydrophilic region (Figure 1(b)). Hexaacylated symmetric lipid A (with three acyl chains bound to the nonreducing and three to the reducing glucosamine) and penta- and tetraacylated lipid A molecules have a cylindrical molecular shape, and they do not have biological activity [16, 17]. The intrinsic conformation of lipid A is not altered when saccharide groups are added, as in LPS. The LPS molecules form multimeric aggregates in water: if the lipid A is cylindrical, they form a smooth bilayer arrangement, but conical lipid A molecules form a nonbilayer or hexagonal (H_II_) arrangement [17]. LPS-binding protein (LBP) is a plasma protein that facilitates the transfer of LPS molecules from these hexagonal (H_II_) arrangements to CD14, and membrane-bound CD14 delivers LPS to TLR-4/MD2 [18]. Since the conical molecular shape of lipid A is a requirement for TLR-4/MD-2 triggering [16–19], we hypothesized that liposomes with nonbilayer phospholipid arrangements, but not smooth liposomes (with phospholipids in a bilayer arrangement), could trigger TLR-4/MD-2 signaling.

In this study, we investigated whether liposomes with nonbilayer phospholipid arrangements are TLR-4/MD-2 agonists, because the activation of this innate immune receptor leads to the production of proinflammatory cytokines. We also looked for proinflammatory cytokines in the sera of mice with the autoimmune disease triggered by liposomes with nonbilayer phospholipid arrangements, and we determined the gene expression profile in the spleens of these mice, focusing on the expression of proinflammatory genes. In addition, we determined the relative percentage and activation of T, NKT, dendritic, and B cells in the spleen of mice with the disease. This study contributes to the understanding of the pathological and genetic features of a novel mouse model of human lupus.

## 2. Materials and Methods

### 2.1. Preparation and Characterization of Liposomes

Egg-yolk L-*α*-phosphatidic acid, bovine brain L-*α*-phosphatidylserine, egg-yolk L-*α*-phosphatidylcholine, chlorpromazine, procainamide, and chloroquine were purchased from Sigma (St. Louis, MO, USA). Liposomes contained the cylindrical shaped phospholipid phosphatidylcholine and a conical phospholipid (phosphatidic acid or phosphatidylserine). The molar ratios (phosphatidylcholine/phosphatidic acid 2 : 1, phosphatidylcholine/phosphatidylserine 4 : 1) were optimized for the induction of nonbilayer phospholipid arrangements [14]. Nine micromoles of phospholipid mixture was dissolved in 1 mL diethyl ether and 330 *μ*L of TS buffer (10 mM Tris-HCl, 1 mM NaCl, pH 7), mixed and sonicated three times in a G112SPI sonicator (Laboratory Supplies, Hicksville, NY, USA). The diethyl ether was then removed under a stream of oxygen-free dry nitrogen at reduced pressure, using a rotary evaporator at 37°C. The liposomes were filtered through 0.45 *μ*m MF-Millipore membranes (Billerica, MA, USA) to homogenize their size.

To induce the formation of nonbilayer phospholipid arrangements, liposomes in TS buffer were incubated for 30 min at 37°C in the presence of 0.5–4 mM MnCl_2_, 0.5–3 mM chlorpromazine, and 4–32 mM procainamide [14]. All of the final preparations of liposomes were negative for LPS contamination, as assessed by the gel clot LAL method (Charles River Endosafe, Charleston, SC, USA).

The detection of nonbilayer phospholipid arrangements by flow cytometry was previously validated by freeze-fracture electron microscopy and ^31^P-NMR spectroscopy [10, 14, 15]. Therefore, in this study we only used flow cytometry to demonstrate the formation of these arrangements on liposomes. Liposomes and liposomes with nonbilayer phospholipid arrangements in TS buffer were analyzed with a FACSCalibur flow cytometer (Becton Dickinson, San Jose, CA, USA) with CellQuest software. Ten thousand events were acquired for each sample.

### 2.2. TLR Activation Assays

Human embryonic kidney (HEK) 293 cells, nontransfected or stably transfected with human TLR-4/MD2/CD14, TLR-2/TLR-6, TLR-5, or TLR-8, were purchased from InvivoGen (San Diego, CA, USA). The expression of the TLRs was verified by flow cytometry. The HEK-TLR transfectants were maintained at 37°C in 5% CO_2_ in Dulbecco's modified Eagle's medium (Invitrogen, Carlsbad, CA, USA) containing 4.5 g/L glucose, 10% heat-inactivated fetal bovine serum (Gibco, Grand Island, NY, USA), 10 *μ*g/mL blasticidin (InvivoGen), and 100 *μ*g/mL normocin (InvivoGen). HygroGold (25 *μ*g/mL; InvivoGen) was also added to the media of the HEK-TLR-4/MD2/CD14 cell line. The viability of these cell lines in the presence of Mn^2+^, chlorpromazine, procainamide, or chloroquine, and in the presence of liposomes or liposomes with nonbilayer phospholipid arrangements, was evaluated with the Alamar Blue method [20].

To assess TLR activation, the cell lines were incubated in the presence of liposomes made of phosphatidylcholine/phosphatidic acid (2 : 1), alone or with nonbilayer phospholipid arrangements induced by Mn^2+^ (2–4 mM). As a negative control, the liposomes with nonbilayer arrangements were previously incubated with 0.1 mM chloroquine. For the positive controls, the cell lines were incubated in the presence of their known TLR agonists: 100 ng/mL* Escherichia coli* 0111:B4 LPS for HEK-TLR-4/MD2/CD14, 1 *μ*g/mL FSL-1 (a synthetic lipoprotein derived from* Mycoplasma salivarium*) for HEK-TLR-2/TLR-6, 1 *μ*g/mL* Salmonella typhimurium* flagellin for HEK-TLR-5, and 2.5 *μ*g/mL ssRNA40 (a 20 mer phosphorothioate-protected single-stranded RNA oligonucleotide containing a GU-rich sequence) for HEK-TLR-8. All TLR agonists were sourced from InvivoGen. After 24 h, the cell culture supernatants were harvested and assayed for IL-8 production (BD OptEIA Set Human IL-8, BD Biosciences, San Diego, CA, USA). NF-*κ*B activation was assayed in cell culture extracts using the reporter plasmid pNiFty-Luc (Promega Corporation, Madison, WI, USA).

In order to determine if chloroquine affects the viability of HEK293 cells, the LIVE/DEAD Fixable Violet Dead Cell Stain Kit (Invitrogen) was used. HEK293 cells were incubated with 0.05, 0.1, and 0.5 mM of chloroquine for 24 h at 37°C and 5% CO_2_. The cells were then transferred to a tube and stained with 50 *μ*L of LIVE/DEAD diluted 1 : 100 in distilled water and incubated for 15 min at room temperature in the dark. FACS lysis buffer (1 mL; Becton Dickinson) was added for erythrocyte lysis, and the cells were incubated for 10 min at room temperature in the dark. The cells were washed with 2 mL of phosphate-buffered saline (PBS) and resuspended in 300 *μ*L of PBS and analyzed by flow cytometry. Forty thousand events were acquired for each sample with a LSR Fortessa cytometer (Becton-Dickinson).

To evaluate whether chloroquine can induce apoptosis of HEK293 cells, the Annexin V-propidium iodide staining method was used. HEK293 cells were incubated with 0.05, 0.1, and 0.5 mM of chloroquine for 24 h at 37°C and 5% CO_2_. The cells were then transferred to a tube and washed with 1 mL of Annexin V-binding buffer (eBioscience, San Diego, CA, USA). 100 *μ*L of 2 *μ*g/mL Annexin V-APC (eBioscience) in Annexin V-binding buffer was added, and the cells were incubated for 15 min at room temperature in the dark. The cells were washed with 1 mL of Annexin V-binding buffer, resuspended in 100 *μ*L of the same buffer containing 1 *μ*g of propidium iodide (BioLegend, San Diego CA, USA) and incubated for 15 min at room temperature in the dark. The cells were washed and resuspended in the Annexin V-binding buffer and analyzed immediately by flow cytometry. Forty thousand events were acquired for each sample in a LSR Fortessa cytometer (Becton-Dickinson).

TLR stimulation was also analyzed in bone marrow-derived macrophages (BMDM) from BALB/c mice. BMDM were obtained from the femur and shinbone of female 2-month-old BALB/c mice and they were cultured in RPMI media with 10% heat-inactivated fetal bovine serum, 50 U/mL penicillin (Gibco), 50 *μ*g/mL streptomycin (Gibco), and 10 ng/mL recombinant M-CSF (BioLegend) for 7 days. For TLR stimulation, the BMDM were cultured in 96-well plates and stimulated with 10 ng/mL LPS, 1 *μ*g/mL peptidoglycan (PGN; InvivoGen), or 10 or 20 *μ*L of smooth liposomes or liposomes bearing nonbilayer phospholipids arrangements, respectively. After incubation for 24 h at 37°C, the supernatants were collected and analyzed by enzyme-linked immunoabsorbent assay (ELISA; BioLegend) for tumor necrosis factor- (TNF-) *α* production. For the blocking experiments, 10 *μ*g/mL of anti-TLR-2 (clone T2.5, BioLegend) or 20 *μ*g/mL of anti-TLR-4 (clone MTS510, BioLegend) was added 2 h before the liposomes. The cells were incubated for 24 h at 37°C and the supernatants were collected and analyzed for TNF-*α*. IgG1,*κ* (clone MOPC-21, BioLegend) and IgG2a,*κ* (clone RTK2758, BioLegend) isotype controls were used for the blocking antibodies.

### 2.3. Mouse Model of Autoimmune Disease Resembling Human Lupus

Forty female 2-month-old specific-pathogen-free BALB/c mice were divided into four groups. The first and the second groups were injected intrasplenically, on days 1 and 15, with phosphatidylcholine/phosphatidic acid (2 : 1) liposomes that had been incubated with 5 mM MnCl_2_ (Mn group) or 3 mM chlorpromazine (CPZ group). Mice received the same amount of liposomes by intraperitoneal injection on day 30 and then every week for 6 months [10]. The negative control groups consisted of 10 mice treated in the same way but using TS buffer alone (Control group I), or liposomes made of phosphatidylcholine/phosphatidic acid (2 : 1) alone (Control group II).

Blood was taken from mice before liposome injection and each month after the first intraperitoneal injection, for a total of 6 months. Sera were heated at 56°C for 30 min to inactivate complement and frozen in aliquots at −70°C. To confirm that these mice developed the disease resembling human lupus, we measured anti-nonbilayer phospholipid arrangements, anti-cardiolipin, anti-histone, and anti-coagulant antibodies in their sera. Anti-nonbilayer phospholipid arrangements antibodies were measured by ELISA where the wells were coated with liposomes with or without nonbilayer phospholipid arrangements [14]. Anti-cardiolipin and anti-histone antibodies were also measured by ELISA. Results are reported as arbitrary units (AU) calculated as (AsP − AsW)/(AsH − AsW), where AsP is the absorbance obtained with the sera of mice injected with the liposomes, AsH is the absorbance obtained with the sera of mice before the injection of liposomes, and AsW is absorbance of controls without sera [10]. A modification of the kaolin-activated thromboplastin time test was used to determine the anti-coagulant antibodies; results are reported as the coagulation time in seconds [21].

Three mice from each of the four groups indicated above were euthanized 4 months after the first injection of nonbilayer phospholipid arrangements, when they had the highest titers of anti-nonbilayer phospholipid arrangements, anti-cardiolipin, anti-histone, and anti-coagulant antibodies, and their spleens were used for gene and protein expression studies. The experimental protocols for animal care and use were reviewed and approved by the Bioethics Committee of our Institution according to the “Guide for the Care and Use of Laboratory Animals,” which was published by the US National Institute of Health [22].

### 2.4. Quantification of Cytokines in Mouse Sera

The serum concentrations of interleukin-6 (IL-6), IL-10, IL-12p70, interferon-*γ* (IFN-*γ*), TNF-*α*, and monocyte chemoattractant protein- (MCP-) 1 were measured with a bead-based multiplex immunoassay (BD CBA Mouse Inflammation Kit). Data were acquired with a FACSCalibur flow cytometer, with CellQuest software.

### 2.5. Evaluation of Gene and Protein Expression in Mouse Spleens

Mouse spleens were sectioned and placed in two cryotubes, one with RNAlater (Invitrogen) for RNA expression studies and one with Tissue-Tek (Sakura Finetek, Torrance, CA, USA) for protein analysis. The cryotubes were stored at −70°C until use. To isolate RNA, the tissue stored in RNAlater was thawed and disaggregated at 15,000 rpm with a TissueRuptor (Qiagen, Valencia, CA, USA), and total RNA was extracted from the tissue homogenates using an RNeasy Mini Kit (Qiagen). The quality and quantity of the RNA samples were assessed in an Agilent BioAnalyzer 2100 (Agilent, Palo Alto, CA, USA) and a NanoDrop 2000 (Thermo Fisher Scientific, Auburn, AL, USA), respectively; only RNA samples with a RNA integrity number (RIN) ≥ 7 were used for the gene expression analysis.

Total RNA (400 ng) was amplified and labeled using the Quick Amp Labeling Kit (Agilent), and the cyanine-3- or cyanine-5-labeled cRNA was purified with an RNeasy Mini Kit (Qiagen). The cRNA were hybridized to 4 × 44 K whole mouse genome microarray chips (Agilent, G4122F); the microarrays were scanned with an Agilent Microarray scanner (G2565BA) and the data were extracted with Agilent Feature Extraction software (v.9.5.3.1). Normalization was performed with GeneSpring GX 11.0 software (Agilent). The cutoff for over- and underexpressed genes was set at a mean fold change log_2_ ratio greater than +2 or lower than −2, as assessed by two-way analysis of variance (ANOVA; Partek Pro software, Partek Inc., St. Charles, MO, USA) with *p* < 0.01 [23].

To evaluate protein expression, the spleen samples stored in Tissue-Tek were thawed, rinsed with PBS, and disaggregated at 15,000 rpm with a TissueRuptor (Qiagen). The homogenates were centrifuged at 5,000 ×g for 5 min at 4°C, and the supernatants were used to measure C3 (ELISA Kit MBS700250, MyBioSource, San Diego, CA, USA), C5 (ELISA Kit MBS704792, MyBioSource), C3a (ELISA Kit MBS70381, MyBioSource), C5a (ELISA Kit MBS700538, MyBioSource), and IFN-*β* (ELISA Kit 439407, BioLegend).

TLR-4 was measured by flow cytometry in cells obtained from fresh spleens, which were disaggregated and passed through a 70 *μ*m nylon mesh. The cells were labeled with a fluorescein isothiocyanate- (FITC-) conjugated anti-F4/80 antibody (BioLegend), a PE-conjugated rat anti-mouse TLR-4 antibody (BioLegend), and Fixable Viability Dye 450 (eBiosciences) and acquired in a FACSCalibur flow cytometer. Single viable cells were analyzed, and the percentage of F4/80^+^ TLR-4^+^ cells of the total live cells was determined.

### 2.6. Evaluation of T, NKT, Dendritic, and B Cells in Mouse Spleens

The spleens of three mice from the groups injected intrasplenically with liposomes without nonbilayer phospholipid arrangements or with liposomes bearing nonbilayer phospholipid arrangements were placed in fluorescence-activated cell sorting (FACS) buffer containing 0.1% BSA and 0.01% sodium azide (Sigma Aldrich). Spleens were disaggregated and passed through a 70 *μ*m nylon mesh. Red blood cells were lysed and spleen cells were resuspended in FACS buffer. Before staining, cells were incubated with Universal Blocking Reagent (Block Biogenex, San Ramón, CA, USA) in PBS for 10 min at 4°C and then washed.

Splenocyte suspensions were labeled with anti-CD19-APC, anti-CD5-AF488, anti-CD69-PerCP, and anti-TLR4-PE (eBioscience) to evaluate B cells; with anti-CD3-FITC, anti-CD4-PE (eBioscience), anti-CD8-APC, and anti-CD69-PerCP to evaluate T cells; with anti-Gr-1-PerCP, anti-CD11c-APC, anti-MHC-II-PE (eBioscience), anti-CD80-FITC, and anti-CD86-PE/Cy7 (eBioscience) to evaluate dendritic cells; and with anti-CD3-FITC and anti-NK 1.1-APC to evaluate NKT cells. Fixable Viability Dye 450 (eBioscience) was added in all cases. The cells were incubated for 30 min at 4°C, then washed with FACS buffer, and fixed with 1% paraformaldehyde (Sigma Aldrich). Labeled cells were acquired in a LSR Fortessa flow cytometer (Becton Dickinson); single, viable cells were analyzed with FlowJo 10.0.6 (Tree Star, Inc., Ashland, OR, USA). Appropriate isotype controls were included in all sets of experiments. All antibodies were from BioLegend unless otherwise indicated.

## 3. Results

### 3.1. Lipopolysaccharide Increases the Complexity of Liposomes

We had previously shown that the presence of nonbilayer phospholipid arrangements can be detected by flow cytometry as an increase in side scatter (SSC) value [10, 14, 15]. Thus, the increase in SSC signal after the addition of Mn^2+^, chlorpromazine, or procainamide to liposomes made of phosphatidylcholine/phosphatidic acid or phosphatidylcholine/phosphatidylserine indicated the presence of nonbilayer phospholipid arrangements (Figures 1(c)-1(d) and 1(f)-1(g)).

As a negative control, we added 5 mM of Mg^2+^ to liposomes (Figures 1(c), 1(d), and 1(e)); Mg^2+^ does not induce the formation of nonbilayer phospholipid arrangements, as was previously shown for phosphatidylcholine/phosphatidic acid liposomes [15]. Liposomes made of the cylindrical lipid phosphatidylcholine, without any conical lipid, did not increase in complexity in the presence of Mn^2+^ or Mg^2+^ (Figure 1(e)), chlorpromazine, or procainamide (data not shown). The addition of LPS caused an increase in SSC signal when it was used alone (Figure 1(h)) or in combination with Mn^2+^ (Figure 1(i)), which suggests that LPS modifies the lipid bilayer. The addition of 0.1 mM chloroquine, a drug that blocks or reverses the formation of nonbilayer phospholipid arrangements [14], decreased the liposome complexity induced by procainamide or Mn^2+^ (Figures 1(g)-1(j)) or chlorpromazine (data not shown).

### 3.2. Liposomes with Mn^2+^-Induced Nonbilayer Phospholipid Arrangements Are Toll-Like Receptor- (TLR-) 2/6 and TLR-4 Agonists

We evaluated the effects of phosphatidylcholine/phosphatidic acid liposomes and phosphatidylcholine/phosphatidylserine liposomes, alone or in the presence of Mn^2+^, chlorpromazine, or procainamide, on the viability of HEK, HEK-TLR-4/MD2/CD14, HEK-TLR-2/TLR-6, HEK-TLR-5, and HEK-TLR-8 cell lines. We found that liposomes made of phosphatidylcholine/phosphatidic acid with Mn^2+^-induced nonbilayer phospholipid arrangements had no effect on the cell viability (90% or more of cells were viable). These liposomes were then used for TLR activation assays.

**Figure 2:**
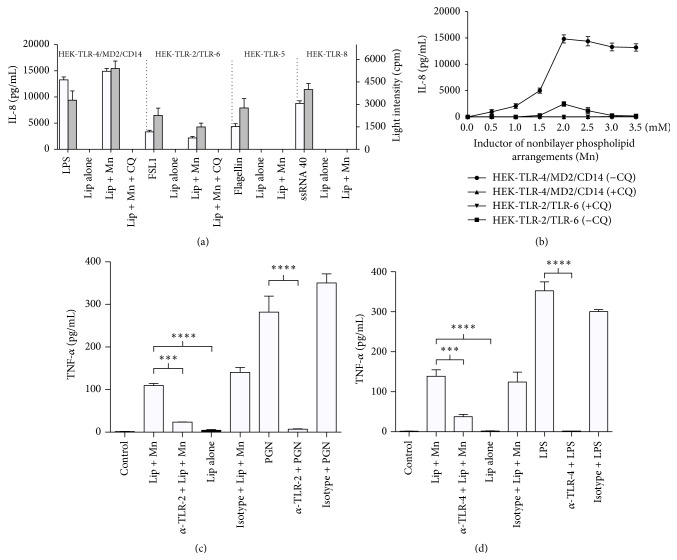
Liposomes with nonbilayer phospholipid arrangements induce TLR-2/6 and TLR-4 signaling in TLR-expressing HEK cells. (a) HEK-TLR-4/MD2/CD14, HEK-TLR-2/TLR-6, HEK-TLR-5, and HEK-TLR-8 cells were stimulated for 24 h at 37°C with 10 *μ*L of egg-yolk phosphatidylcholine/egg-yolk phosphatidic acid (2 : 1) liposomes (50 nmol phosphatidic acid in 50 *μ*L TS buffer) alone or bearing nonbilayer phospholipid arrangements induced with 2 mM MnCl_2_. As positive controls, cells were stimulated with their TLR agonist: 100 ng/mL LPS, 1 *μ*g/mL FSL1, 1 *μ*g/mL bacterial flagellin, or 2.5 *μ*g/mL ssRNA40, respectively. As negative controls, cells were stimulated with liposomes alone or bearing nonbilayer phospholipid arrangements + 0.1 mM chloroquine (CQ). IL-8 levels and NF-*κ*B activation were measured 24 h after stimulation. (b) HEK-TLR-4/MD2/CD14 and HEK-TLR-2/TLR-6 cells were stimulated with nonbilayer phospholipid arrangements induced with different MnCl_2_ concentrations with (+CQ) or (−CQ) 0.1 mM chloroquine. IL-8 levels were measured 24 h after stimulation. Histograms and graphs represent the mean ± SD of three independent experiments. (c, d) TLR-2 and TLR-4 of bone marrow-derived macrophages (BMDM) were analyzed. (c) 4 × 10^4^ BMDM were stimulated with liposomes alone or bearing Mn^2+^-induced nonbilayer phospholipid arrangements (as in a). Where indicated, 10 *μ*g/mL of anti-TLR-2 or isotype control (IgG1,*κ*) was added 2 h before liposomes addition. PGN (1 *μ*g/mL) was the positive control. After 24 h at 37°C, TNF-*α* levels were determined. (d) 3 × 10^3^ BMDM were stimulated with 20 *μ*L of liposomes (as in a). Where indicated, 20 *μ*g/mL of anti-TLR-4 or isotype control (IgG2a,*κ*) was added 2 h before liposomes addition. LPS (10 ng/mL) was the positive control. TNF-*α* levels were determined as in (c). Histograms represent the mean ± SD of three independent experiments. Data were analyzed with one-way ANOVA and Tukey's multiple comparisons test. ^*∗∗∗∗*^ *p* ≤ 0.0001, ^*∗∗∗*^ *p* ≤ 0.001.

Liposomes with Mn^2+^-induced nonbilayer phospholipid arrangements stimulated IL-8 production by HEK-TLR-4/MD2/CD14 cells and, to a lesser degree, by HEK-TLR-2/TLR-6 cells, but not by HEK-TLR-5 or HEK-TLR-8 cells (Figure 2(a)). Liposomes without nonbilayer phospholipid arrangements or liposomes in which Mn^2+^-induced nonbilayer phospholipid arrangements had been reversed by chloroquine did not induce IL-8 production (Figure 2(a)). Similar results were obtained when NF-*κ*B activation was measured through the reporter plasmid pNiFty-Luc (Figure 2(a)). Nontransfected HEK cells did not produce IL-8 in the presence of liposomes with Mn^2+^-induced nonbilayer phospholipid arrangements (data not shown).

The production of IL-8 by HEK-TLR-4/MD2/CD14 or HEK-TLR-2/TLR-6 cells in response to liposomes with Mn^2+^-induced nonbilayer phospholipid arrangements was dose-dependent, and the effect was inhibited by chloroquine (Figure 2(b)). Cell viability in the presence of chloroquine was 90% or higher, and chloroquine did not induce apoptosis of these cells at the tested concentrations (see Supplementary Figure 1 in Supplementary Material available online at http://dx.doi.org/10.1155/2015/369462). Thus, the effects observed in the presence of chloroquine can be attributed to a reversion of Mn^2+^-induced nonbilayer phospholipid arrangements by this drug.

Additionally, we found that nonbilayer phospholipid arrangements induce the production of the proinflammatory cytokine TNF-*α* by BMDM from BALB/c mice. The production of TNF-*α* induced by smooth liposomes was significantly lower. Furthermore, anti-TLR-2 and anti-TLR-4 antibodies blocked the production of TNF-*α* by BMDM in response to nonbilayer phospholipid arrangements (Figures 2(c)-2(d)).

### 3.3. Proinflammatory Cytokines Are Found in the Sera of Mice with a Disease Resembling Human Lupus

**Figure 3:**
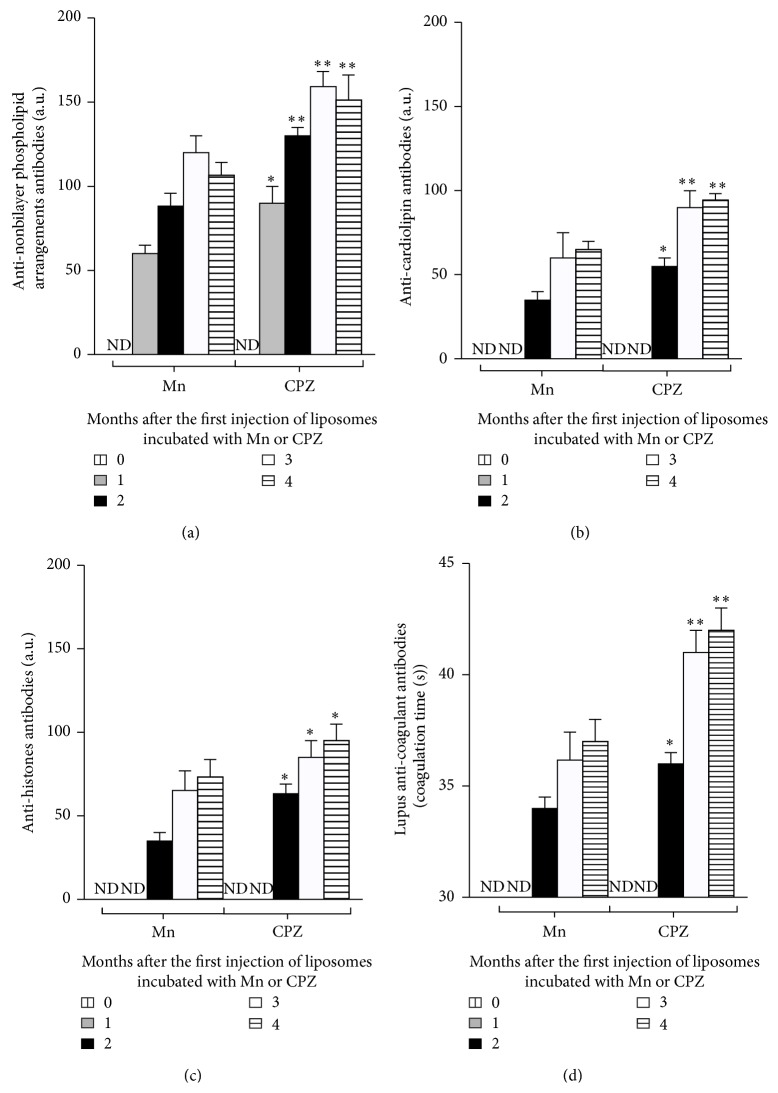
Antibodies found in the sera of mice with the disease resembling human lupus. Mice (10 per group) were injected with egg-yolk phosphatidylcholine/egg-yolk phosphatidic acid (2 : 1) liposomes (50 nmol phosphatidic acid in 50 *μ*L TS buffer), alone (negative control) or incubated 30 min at 37°C with 5 mM MnCl_2_ or 3 mM chlorpromazine (CPZ) to induce nonbilayer phospholipid arrangements. Mice injected with TS buffer were also used as negative control. (a) Anti-nonbilayer phospholipid arrangements, (b) anti-cardiolipin (CL), (c) anti-histone, and (d) lupus anti-coagulant antibodies were measured before and 1–4 months after liposome injection. Histograms represent the mean ± SD of three independent experiments. Control mice injected with TS buffer or with liposomes without nonbilayer phospholipid arrangements did not have detectable levels of any of these four antibodies, 1 or 4 months after the administration of TS or liposomes. ND: not detected. Data were analyzed with Kruskal-Wallis test with Dunn's post-test (GraphPad Prism). ^*∗*^ *p* < 0.05; ^*∗∗*^ *p* < 0.01. Asterisks indicate statistical significance between the antibody titers induced by liposomes incubated with CPZ and those incubated with Mn.

Liposomes with nonbilayer phospholipid arrangements induced by Mn^2+^ or chlorpromazine were used to produce an autoimmune disease resembling human lupus in mice. Antibodies against nonbilayer phospholipid arrangements were detected 1 month after the first injection of liposomes with nonbilayer phospholipid arrangements, and the titers in mice injected with chlorpromazine-induced nonbilayer phospholipid arrangements were higher than in those injected with Mn^2+^-induced nonbilayer phospholipid arrangements (*p* < 0.001). These antibodies appeared 1 month before the anti-cardiolipin, anti-histone, and anti-coagulant antibodies (Figures 3(a), 3(b), 3(c), and 3(d)). The presence of the four autoantibodies confirmed that the disease had been developed in the mice. Control mice injected with TS buffer or with liposomes without nonbilayer phospholipid arrangements did not generate any of the four autoantibodies.

**Figure 4:**
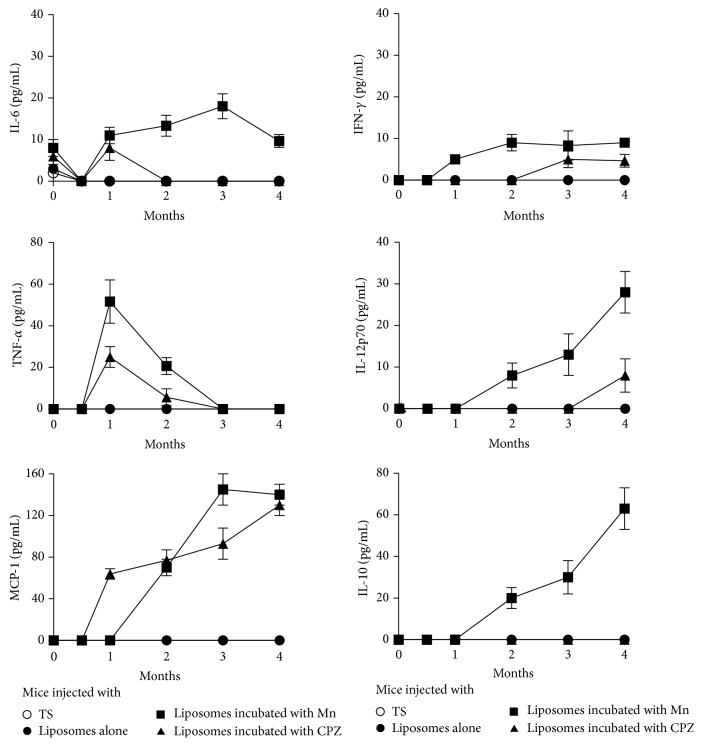
Proinflammatory cytokines are found in the sera of mice with the disease resembling human lupus. Cytokines were detected in the sera of mice injected with egg-yolk phosphatidylcholine/egg-yolk phosphatidic acid (2 : 1) liposomes (50 nmol phosphatidic acid in 50 *μ*L TS buffer) incubated with 5 mM MnCl_2_ or 3 mM chlorpromazine (CPZ). Months indicate the time after the first injection of liposomes. The detection limits for each cytokine were 5 pg/mL IL-6, 7.3 pg/mL TNF-*α*, 52.7 pg/mL MCP-1, 2.5 pg/mL IFN-*γ*, 10 pg/mL IL-12p70, and 17.5 pg/mL IL-10. Control groups (mice injected with TS buffer or with liposomes without nonbilayer phospholipid arrangements) did not have detectable levels of these cytokines. Bars represent the mean ± SD of three independent experiments with six mice each.

IL-6, IL-10, IL-12p70, IFN-*γ*, TNF-*α*, and MCP-1 were found in the sera of mice injected with liposomes with Mn^2+^-induced nonbilayer phospholipid arrangements; IL-6, IFN-*γ*, and TNF-*α* appeared 1 month after treatment, while IL-10, IL-12p70, and MCP-1 were found after 2 months. These cytokines were also found in the sera of mice injected with chlorpromazine-induced nonbilayer phospholipid arrangements; IL-6, TNF-*α*, and MCP-1 appeared 1 month after treatment, while IFN-*γ* and IL-12p70 were found 4 months after treatment. None of the tested cytokines were found in the sera of mice treated with smooth liposomes (Figure 4).

### 3.4. C3, C5, TLR-4, and TLR-4-Signaling Molecules and IFN-*β* Are Overexpressed in the Spleens of Mice with an Autoimmune Disease Resembling Human Lupus

We evaluated gene expression in the spleens of mice from the four treatment groups: group 1, mice injected with TS buffer alone (Control I); group 2, mice injected with smooth liposomes (liposomes without nonbilayer phospholipid arrangements, Control II); group 3, mice that received liposomes with Mn^2+^-induced nonbilayer phospholipid arrangements (Mn group); and group 4, mice that received liposomes with chlorpromazine-induced nonbilayer phospholipid arrangements (CPZ group). Spleens were collected 4 months after treatment, and the cRNA derived from the spleens of three mice from each group were pooled and hybridized to a whole mouse genome microarray chip.

No significant differences were found between Control I and Control II groups; 426 genes were overexpressed and 62 genes were underexpressed in the Mn group, compared with the Control II group; 542 genes were overexpressed and 73 genes were underexpressed in the CPZ group, compared with the Control II group; and 383 genes were overexpressed and 44 genes were underexpressed in the CPZ group, compared with the Mn group.  Table 1 shows a list of genes that were overexpressed in both the Mn and CPZ groups, compared with the Control II group. This includes genes for complement components (C3 and C5), molecules involved in the presentation of exogenous antigens, in the production of antibodies, and in TLR-4 and NOD-2 signaling.  Table 1 also shows a list of genes that were underexpressed in both the Mn and CPZ groups, compared with the Control II group. These are genes for molecules that are involved in apoptosis and in NK cell recognition.

**Figure 5:**
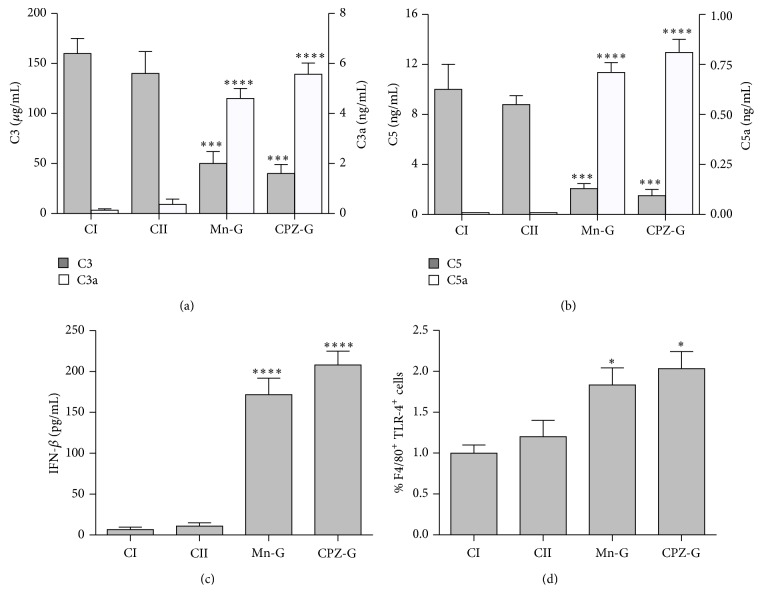
C3, C5, TLR-4, and IFN-*β* are overexpressed in the spleens of mice with a disease resembling human lupus. The complement proteins C3 and C5 (a), the fragments C3a and C5a (b) (produced by C3 and C5 cleavage), and IFN-*β* (c) were detected in a cell-free extract from the spleen of mice injected with TS buffer, Control I group (CI), egg-yolk phosphatidylcholine/egg-yolk phosphatidic acid (50 nmol phosphatidic acid in 50 *μ*L TS) liposomes alone, Control II group (CII), or bearing Mn^2+^-induced nonbilayer phospholipid arrangements (Mn-G) or chlorpromazine-induced nonbilayer phospholipid arrangements (CPZ-G). The detection ranges were C3 (4.69–300 *μ*g/mL), C3a (0.16–10 ng/mL), C5 (0.25–16 ng/mL), C5a (0.062–4.0 ng/mL), and IFN-*β* (7.8–500 pg/mL). TLR-4 (d) was detected by flow cytometry on cells obtained from fresh spleens from the four treatment groups. Bars represent the mean ± SD of three independent experiments, with three mice in each of the four groups. Data were analyzed with one-way ANOVA followed by Tukey's multiple comparisons test. ^*∗∗∗∗*^ *p* ≤ 0.0001, ^*∗∗∗*^ *p* ≤ 0.001, and ^*∗*^ *p* ≤ 0.05. Asterisks indicate statistical significance between the control groups and the Mn or CPZ groups.

The C3 and C5 complement proteins were increased in the Control I and Control II groups, compared with the Mn and CPZ groups. However, C3a and C5a, two active fragments that are produced by C3 and C5 cleavage, were increased in the Mn and the CPZ groups, compared with the Control I and Control II groups (Figures 5(a)-5(b)). IFN-*β* was also increased in the spleens of mice with the autoimmune disease, compared with healthy mice (Figure 5(c)). The number of cells expressing TLR-4 increased in the Mn and the CPZ groups, compared with the Control I and Control II groups (Figure 5(d)).

### 3.5. NKT, Dendritic, and B Cells Are Increased in the Spleens of Mice with an Autoimmune Disease Resembling Human Lupus

**Figure 6:**
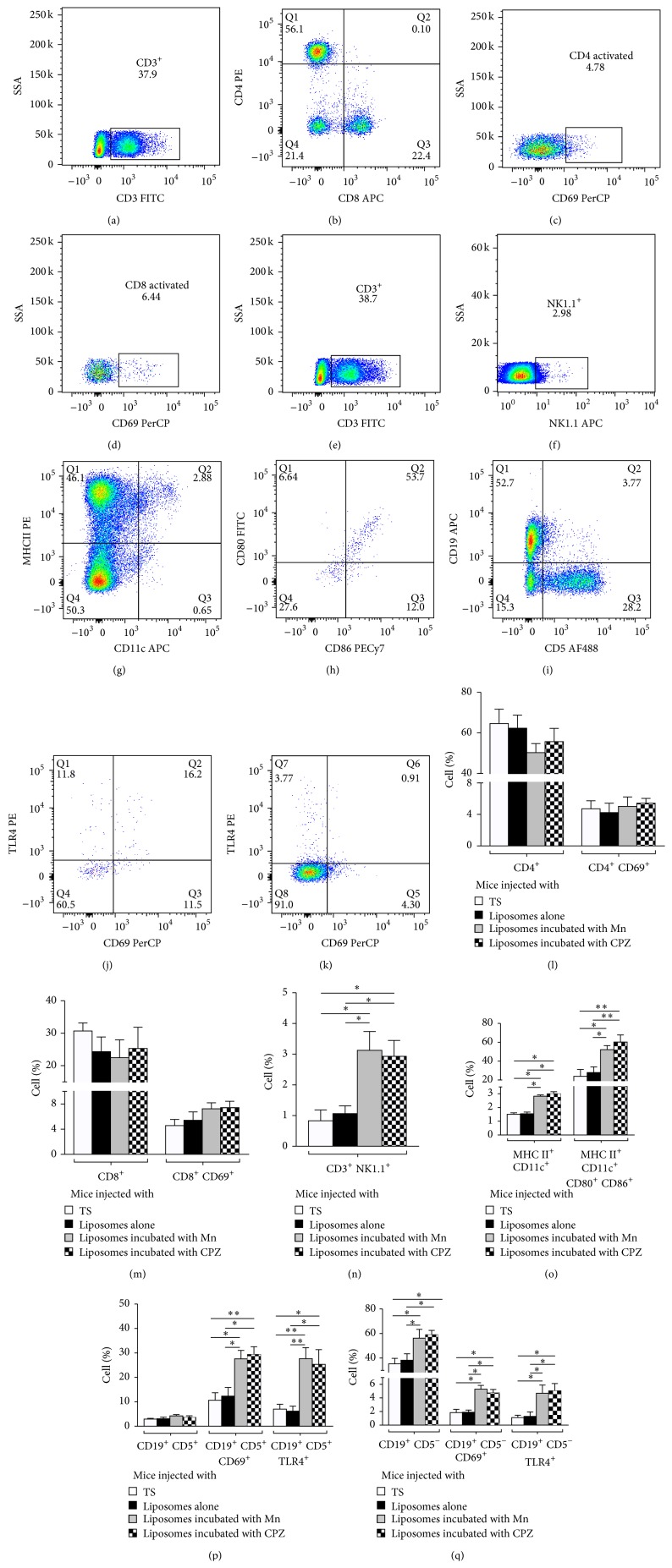
Dendritic, B1, and B2 cells are activated in mice with an autoimmune disease resembling human lupus. To analyze the percentage and activation of immune cells, cell suspensions from the spleens of mice injected with TS buffer, liposomes alone, or liposomes incubated with Mn or CPZ were labeled with antibodies and analyzed by flow cytometry. Gating strategy for the identification of activated CD4 (CD3^+^, CD4^+^, CD8^−^, and CD69^+^) (a–c) and CD8 (CD3^+^, CD4^−^, CD8^+^, and CD69^+^) (a, b, and d) T cells; NKT cells (CD3^+^, NK1.1^+^) (e, f); activated dendritic cells (MHCII^+^, CD11c^++^, CD80^+^, and CD86^+^) (g, h); activated B1 (CD19^+^, CD5^+^, and CD69^+^) and B2 (CD19^+^, CD5^−^, and CD69^+^) cells; and expression of TLR-4 (i–k). Percentage of total NKT (n); total and activated CD4 (l) and CD8 (m) T cells, dendritic cells (o), and B1 (p) and B2 (q) cells. The expression of TLR-4 was evaluated on B1 (p) and B2 (q) cells. Kruskal-Wallis test with Dunn's post-test was used for statistical analysis; significance was set at *p* < 0.05. Asterisks represent statistically significant differences between the indicated groups (^*∗*^ *p* < 0.05, ^*∗∗*^ *p* < 0.01).

Activated CD4 and CD8 T cells (Figures 6(a), 6(b), 6(c), and 6(d)), NKT cells (Figures 6(e)-6(f)), activated dendritic cells (Figures 6(g)-6(h)), and activated and TLR4 expressing B1 and B2 cells (Figures 6(i), 6(j), and 6(k)) were identified by flow cytometry in the spleens of mice. Fifteen days after the mice were injected intrasplenically with liposomes bearing nonbilayer phospholipids arrangements induced by Mn or chlorpromazine, the percentage and activation of CD4 and CD8 T cells were not increased, compared with the control mice that received TS buffer or liposomes alone (Figures 6(l)-6(m)). In contrast, the percentage of NKT, dendritic, and B2 cells was increased (Figures 6(n), 6(o), and 6(q)), and the activation of dendritic and B2 cells was also increased (Figures 6(o)-6(q)). An increase in TLR4 expression was also observed in B2 cells (Figure 6(q)). B1 cells did not increase in percentage, but the number of activated and TLR4 expressing B1 cells did increase (Figure 6(p)).

## 4. Discussion

SLE is a systemic autoimmune disease of unknown etiology characterized by B and T cell hyperactivity, by defects in the clearance of apoptotic cells and immune complexes, and by production of a complex mixture of various cytokines, chemokines, signaling molecules, and pattern-recognition receptors involved in immunity [4, 24]. We have previously demonstrated that liposomes with nonbilayer phospholipid arrangements trigger a disease that resembles human lupus in mice and that IgM and IgG specific to nonbilayer phospholipid arrangements are produced in these mice. Now, we demonstrate that nonbilayer phospholipid arrangements are agonists for TLR-4/MD-2. The activation of this innate immune receptor leads to the production of proinflammatory cytokines; a proinflammatory environment is needed for efficient activation of the adaptive immune response and the production of IgG antibodies. These findings were supported by the increase in the percentage of NKT cells and by the increase in the percentage and activation of dendritic and B2 cells. In addition, the activation of TLR-4/MD2/CD14 by liposomes with Mn^2+^-induced nonbilayer phospholipid arrangements supports our hypothesis on the similarity of the structure of conical phospholipids, which form an inverted micelle inside the nonbilayer arrangement, with the conical association of the acyl chains of the lipid A moiety of LPS.

The importance of the lipid A moiety of LPS was taken into account in the design of glucopyranosyl lipid A (GLA), a synthetic lipid A with six acyl chains and a single phosphate group. GLA as a stable oil-in-water-emulsion (GLA-SE) is a TLR-4 agonist, which signals through MyD88 and TRIF and drives a polyclonal T_H_1 response* in vivo*, characterized by IFN-*γ*, TNF-*α*, and IL-2 producing cells and IgG2c isotype switching [25, 26].

We performed our TLR activation assays in HEK cells transfected with various human TLRs. Interestingly, we also found that nonbilayer phospholipid arrangements induce the production of the proinflammatory cytokine TNF-*α* by BALB/c mouse BMDM. Furthermore, anti-TLR-2 and anti-TLR-4 antibodies blocked the production of TNF-*α* by these macrophages in response to nonbilayer phospholipid arrangements. These findings confirmed our observations with the HEK cells transfected with human TLRs, which also showed that nonbilayer phospholipid arrangements are agonists for TLR-4/MD-2 and TLR-2/TLR-6.

We observed that liposomes with nonbilayer phospholipid arrangements were agonists for TLR-2/TLR-6, but the activation was 3-fold lower than for TLR-4/MD2/CD14. Bacterial macroamphiphilic molecules, such as lipoproteins (including the synthetic lipoprotein FSL-1), lipoteichoic acids, lipoglycans, glycolipids, and lipoarabinomannans, are anchored on bacterial envelopes through a lipidic structure, which is usually a diacylglyceryl moiety. These amphiphilic molecules are mainly recognized via their lipid anchor through TLR-2, alone or as a heterodimer with TLR-1 or TLR-6 [27, 28]. Because the liposomes bearing nonbilayer phospholipid arrangements are made of phosphatidylcholine and phosphatidate, which also have the diacylglyceryl moiety, it is possible that this lipid moiety activated the TLR-2/TLR-6 heterodimer.

TLRs not only recognize pathogen-associated molecular patterns, such as LPS, but also recognize damage-associated molecular patterns, which are released by cells that are either under stress or undergoing apoptosis or necrosis [29]. Examples of damage-associated molecular patterns that are TLR-4 agonists include heat-shock protein 60, fibronectin, fibrinogen, *α*-defensins, and hyaluronan. The molecular structure of these agonists is different from that of LPS, but they all have hydrophobic regions, which are probably recognized by TLR-4 [30]. The modification of the lipid bilayer of cell membranes could be a signal of cell stress: nonbilayer phospholipid arrangements are normally transitory, but if they are stabilized by Mn^2+^ or by the drugs chlorpromazine or procainamide, they could activate the innate immune response via TLRs and then induce the production of antibodies, with the subsequent development of an autoimmune disease.

TLR-4 signaling leads to the activation of NF-*κ*B and the production of proinflammatory cytokines, including TNF-*α*, IL-12, and IFN-*γ*, and chemokines, such as MCP-1. We found these cytokines and chemokines in the sera of mice treated with liposomes with Mn^2+^- or chlorpromazine-induced nonbilayer phospholipid arrangements. The increase in the concentration of the proinflammatory cytokines IL-6 and TNF-*α* correlated with the appearance of anti-nonbilayer phospholipid arrangement antibodies 1 month after the first injection of mice with nonbilayer phospholipid arrangements, and this also corresponds to the period of disease onset. The chemokine MCP-1 and the proinflammatory cytokines INF-*γ* and IL-12p70 increased between months 2 and 4 and correlated with the development and establishment of the disease, given by an increase in the titers of anti-nonbilayer phospholipid arrangement antibodies and the presence of anti-cardiolipin, anti-histone, and anti-coagulant antibodies. IL-10 was only detected in mice that received Mn^2+^-induced nonbilayer phospholipid arrangements. The proinflammatory cytokines IL-1, IL-6, IFN-*γ*, and TNF-*α* and the immunomodulatory cytokines IL-10 and tumor growth factor-*β* (TGF-*β*) have been identified as important players in the development of SLE [31, 32]. The cytokine pattern that we report indicates another similarity of this mouse model with the human disease.

TLR-4 was increased at the mRNA level and the number of cells that express TLR-4 increased in the spleens of mice that received liposomes with nonbilayer phospholipid arrangements. Other genes associated with TLR-4 signaling, such as* Tram*,* Trif*,* Tbk1*, and* Irf3*, were also increased at the mRNA level in these mice. These genes are associated with TRIF-dependent, but not MyD88-dependent, TLR-4 signaling [33]. TRIF-dependent TLR-4 signaling leads to the production of IFN*-α* and *β*. IFN-*β* was increased in the spleens of mice that had received liposomes with nonbilayer phospholipid arrangements compared with healthy mice, and increased levels of IFN*-α* and *β* are reported in patients with SLE [34].

The expression of genes associated with the classical pathway of complement activation (*C1ra*,* C1s*,* C1q*,* C3*,* C5*, and* C7*) was increased in mice that had received liposomes with nonbilayer phospholipid arrangements, and this mRNA increase correlated with the detection of C3a and C5a proteins in the spleens of mice with the autoimmune disease. Complement has an important role in the immune response, but it also has the potential to cause tissue damage, as has been reported in SLE and other autoimmune diseases [35, 36]. It will be interesting to evaluate the role of complement in the tissue damage that is observed in this mouse model of autoimmune disease.

In contrast, the expression of genes associated with NK cell activation (*Klrb1a*,* Klrb1c*,* Klra23*,* Klra7*,* Gzmb*, and* Klra22*) was decreased in mice that received liposomes with nonbilayer phospholipid arrangements. This decrease could reflect a reduction in the absolute number of NK cells or a lower activation of the existing NK cells. The expression of genes associated with apoptosis (*Casp8*,* Cycs*,* Apaf1*, and* Aaifm1*) was also decreased in mice that received liposomes with nonbilayer phospholipid arrangements. This could be relevant for disease development, since deficient apoptosis could favor the survival of autoreactive T cells.

An important additional support for our hypothesis on the effect of nonbilayer phospholipid arrangements on the innate immune response is our finding that mice with the autoimmune disease resembling human lupus have an increase in NKT and dendritic cell percentages, together with increased dendritic cell activation. These cells could recruit and activate B1 and B2 cells, which are the precursors of plasma cells that produce antibodies against nonbilayer phospholipid arrangements.

## 5. Conclusions

The findings reported in this paper are consistent with a mouse model in which nonbilayer phospholipid arrangements directly activate TLR-4 and TLR-2/TLR-6 and lead to the production of proinflammatory cytokines. The proinflammatory environment leads to the efficient activation of the adaptive immune response to the production of IgG antibodies specific for nonbilayer phospholipid arrangements. These antibodies bind to the nonbilayer phospholipid arrangements that are transitorily formed on the surface of many cells and cause cell lysis; the exposure of intracellular antigens could then lead to the formation of anti-cardiolipin, anti-histone, and anti-coagulant antibodies. Furthermore, the inflammatory environment can cause complement-mediated tissue damage and IFN-*β* production. Thus, this mouse model of autoimmune disease recapitulates many features of human lupus.

## Supplementary Material

The Supplementary Material shows that chloroquine has no effect on the viability of HEK-TLR cells (ViViD staining, flow cytometry), and it does not induce apoptosis in these cells (annexin V and propidium iodide, flow cytometry).

## Figures and Tables

**Table 1:** Gene expression in mice with a disease resembling human lupus triggered by liposomes with Mn^2+^-induced or chlorpromazine-induced nonbilayer phospholipid arrangements.

	Genes
Pathways with overexpression of genes	
Complement system	Classical pathway: *C1ra*, *C1s*, *C1q*, *C3*, *C5*, and *C7*
	Receptors of the classical pathway: *C3ar1 *and *C5ar1*
	Alternative pathway: *Cfd*, *Cfh*, and *Cfhr2*
Exogenous antigen presentation	*H2-aa*, *H-2aa*, *H2-dma*, and *Clip*
Antibody production	*Igh-vj558*, *Lyn*, *Syk*, *Plcg2*, *Can*, *Akt1*, and *Nfkb1*
TLR-4 signalling	*Tlr-4*, *Tram*, *Trif*, *Tbk1*, *Irf3*, *Ifn-α*, and *Ifn-β*
NOD-2 signalling	*Nod-2*, *Ripk2*, *Card9*, *Mapk10*, and *Tnfa*
Pathways with underexpression of genes	
Apoptosis	*Casp8*, *Cycs*, *Apaf1*, and *Aifm1*
Recognition of NK cells	*Klrb1a*, *Klrb1c*, *Klra23*, *Klra7*, *Gzmb*, and *Klra22*

Genes that were over- or underexpressed in mice injected with Mn-induced nonbilayer phospholipid arrangements (Mn group) or chlorpromazine-induced nonbilayer phospholipid arrangements (CPZ group), compared with mice injected with liposomes without nonbilayer phospholipid arrangements (Control II). The cutoff for over- and underexpressed genes was set as mean fold change log_2_ ratio greater than +2 or lower than −2, as assessed by two-way ANOVA, with *p* < 0.01.
