# Supplementary material for: Nonbilayer Phospholipid Arrangements Are Toll-Like Receptor-2/6 and TLR-4 Agonists and Trigger Inflammation in a Mouse Model Resembling Human Lupus
Source: J Immunol Res. 2015 Oct 19;2015:369462. doi: 10.1155/2015/369462 (PMC4629040; doi:10.1155/2015/369462)
Supplement: Supplementary file 1 — The Supplementary Material shows that chloroquine has no effect on the viability of HEK-TLR cells (ViViD staining, flow cytometry), and it does not induce apoptosis in these cells (annexin V and propidium iodide, flow cytometry). [file 369462.f1.pdf]

Supplementary figure 1

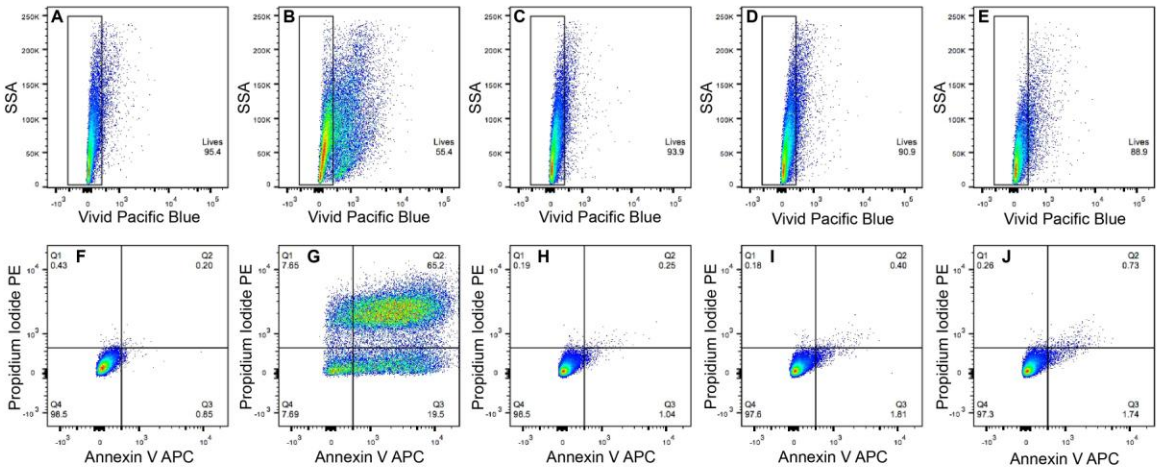

Supplementary figure 1. Chloroquine did not affect the viability or induce apoptosis in HEK-TLR cells. HEK-TLR cells alone (A) and cells with 510 nM dexamethasone (B) or with 0.05 (C), 0.1 (D) or 0.5 (E) mM CQ were stained with ViViD to analyze their viability by flow cytometry. Cells alone (F) and cells with 510 nM dexamethasone (G) or with 0.05 (H), 0.1 (I) or 0.5 (J) mM CQ were stained with annexin V and propidium iodide to analyze apoptosis by flow cytometry.
